# Active involvement of children aged 11–12 years in the development of a healthy nutrition intervention – a qualitative evaluation from researchers’ and children’s perspectives

**DOI:** 10.1186/s12889-025-24019-x

**Published:** 2025-08-14

**Authors:** Hannah Jilani, Imke Schilling, Ansgar Gerhardus, Urte Klink

**Affiliations:** Department for Health Services Research, Institute for Public Health and Nursing Research– IPP, University of Bremen, Grazer Str. 4, Bremen, D-28359 Germany

**Keywords:** Children, Adolescents, Active involvement, Evaluation, Health research, Nutrition

## Abstract

**Background:**

The active involvement of target groups is gaining increasing recognition in health research. Children, in particular, have unique needs and perspectives that differ significantly from those of adult researchers, making their participation essential for improving the relevance and quality of research. Moreover, actively involving children fosters their empowerment. To advance this field, the generation of new knowledge and the refinement of methodologies are crucial. While previous studies have often engaged children with specific health conditions there remains a lack of research evaluating the involvement of healthy children, particularly in terms of process. Therefore, this study aims at answering the two following research questions: (1) What are the barriers and facilitators from the researchers’ perspective when involving healthy children in research? and (2) How do children perceive their active involvement in developing a healthy nutrition intervention in Germany?

**Methods:**

From September 2023 to January 2024, an intervention to promote healthy nutrition was collaboratively developed with 15 healthy children aged 11 to 12 years and two researchers as part of a school activity group. The process began with joint discussions to clarify goals and roles. Intervention components were developed collaboratively with the children. Researchers documented the process through personal minutes and post scripts, which were analysed to identify barriers and facilitators across three phases: ‘Initiating the process’, ‘Building relationships and defining aims’, and ‘Collaboration process’. Additionally, a child-friendly, semi-structured focus group discussion was conducted to evaluate the process, with the data analysed narratively.

**Results:**

The researchers’ protocols and postscripts identified both barriers and facilitators across all phases. Overall, the process demanded considerable openness and flexibility from both children and researchers. Establishing shared goals and clarifying roles was time-consuming, and balancing differing expectations without causing disappointment to any of the group proved challenging. Nevertheless, the focus group showed that children enjoyed the project, appreciated the relaxed after-school setting, and valued the interactive approach. They reported learning a lot but expressed disappointment over the limited hands-on activities, such as food preparation, which they had anticipated.

**Conclusions:**

The evaluation of children’s active involvement highlighted the importance of clear expectation management for successful collaboration. This requires time, openness, and flexibility from all participants. The findings provide practical insights for researchers, supporting the effective involvement of children while reducing potential barriers in future projects.

**Supplementary Information:**

The online version contains supplementary material available at 10.1186/s12889-025-24019-x.

## Background

In health research, the active involvement of the target population has increasingly been recognized as essential by researchers, funders, and the public (e.g [1, 2]). One definition of active involvement was proposed by INVOLVE [3] and used in various publications: ‘Research that is conducted with or by members of the public rather than to, about or for the public’ [4, 5]. Such involvement can improve research quality by ensuring greater relevance to the target group’s needs [6], enhancing participant recruitment, and supporting more effective dissemination [7–9]. Beyond quality, active involvement also promotes empowerment and satisfaction among those involved [7, 9–11].

Active involvement starts with the planning of the process thoroughly and can be challenging for both researchers and participants, especially when undertaken for the first time. Active involvement is possible at every stage of the research process [4, 8, 12]. While involving the target group throughout the entire project is considered most impactful, meaningful involvement at specific stages of the research process can also be effective [13]. Likewise, the intensity of involvement can vary. One way to describe intensity of involvement was suggested by Oliver et al. as a continuum from consultation (low intensity) over collaboration (middle intensity) to research led by the target group (high intensity) [14, 15].

Given the benefits of active involvement, it is especially important to include vulnerable groups in research. Children, as minors, are particularly vulnerable. They rely on caregivers and cannot provide independent consent. As a result, parents or legal guardians are often involved as proxies in the research process [16]. However, given their unique perspectives, ways of thinking, communicating, and behaviour, children should be actively involved in research that directly affects their lives [17, 18]. Children should be actively involved in research that affects their lives, as their needs, perspectives, and experiences often differ from those of adults. Without this understanding, research risks lacking relevance to children’s realities. Active involvement can enhance both relevance and impact. In the UK, several guidelines outline the importance of involving children, key considerations, and methods for meaningful collaboration throughout the research process [19–22]. The importance of actively involving children from an ethical perspective was also highlighted by ‘The International Charter for Ethical Research Involving Children’ [23]. Research that used the method of Participatory Action Research to engage with children aged 7 to 12 showed that children can provide valuable information about food choice motives for future public health (research) projects [24]. Recognising the value of active involvement does not automatically translate into knowing how to implement it. Documented experiences and target group–specific recommendations can support both researchers and participants in successfully initiating participatory projects.

A scoping review focussing on participatory studies that involved children and adolescents into the development of health and well-being interventions included 41 studies [16]. Most of these studies focused on specific health conditions such as asthma, cancer, and diabetes. A recent systematic review also examined the methods used to involve children and adolescents in developing health resources and interventions across 26 studies [25]. Reported participatory methods included workshops, focus groups, and interviews. The review also identified enablers and barriers to successful involvement. Some were specific to the study context (e.g. timelines, ethical issues), others more general, such as the need for flexibility and addressing power imbalances. Notably, neither review included studies from Germany or other German-speaking countries, and most focused on children with specific health conditions [25].

Therefore, this study involved healthy children aged between 11 and 12 years in a two-phase research project. In phase one, children were invited to join the research team for the duration of the project, participating in weekly school-based meetings to develop an intervention aimed at improving dietary behaviour in healthy children. The second phase focussed on the implementation and evaluation of the developed school-based intervention and will be described elsewhere [Klink U, Jilani H. Implementation and Evaluation of a Co-Developed Intervention to Promote Healthy Eating: Designed by Children for Children Journal of Prevention, Submitted.]. This article focuses on the first phase of the project, the intervention development, and addresses two research questions: (1) What are the barriers and facilitators from the researchers’ perspective when involving healthy children in research? and (2) How do children perceive their active involvement in developing a healthy nutrition intervention in Germany? The findings are intended to provide valuable insights for researchers planning to actively involve this population and to support the practical implementation of child involvement by identifying facilitators and barriers.

## Methods

There are several definitions of participatory research and the active involvement of the target group. In this project, we followed the definition provided by INVOLVE, as described in the background section (‘Research that is conducted with or by members of the public rather than to, about or for the public’). With regard to the levels of active involvement, ranging from consultation to collaboration to user-led, we aimed for collaboration. In practice meaning that we aimed to develop the intervention together as a team of researchers and children. For the sake of continuity, we use active involvement as an overarching term, and refer to the shared activities within the process as collaboration or a collaborative process.

### Setting for involvement

The project began in September 2023 at a comprehensive secondary school in Bremen, Germany, located in a low- socioeconomically and ethnically diverse neighbourhood. The aim was to actively involve children (the target group) in developing an intervention to promote healthier dietary behaviour. Following personal meetings with the headmaster, the school administration received detailed study information and provided written informed consent. Ethical approval was granted by the University of Bremen’s institutional review board, confirming adherence to the Declaration of Helsinki.

### Participants and recruitment

The selected school is attended by children from the age of ten (fifth grade), but only sixth-grade students (aged 11–12) were eligible to join after-school activity groups. At the start of the 2023/2024 school year, all sixth-grade students were required to choose one such group. A ‘nutrition’ group, which functioned as our research group, was offered alongside other non-study-related groups. Fifteen children chose to join the nutrition group.

During the first session, children received a detailed study information and a consent form to be signed by their parents. Participation was voluntary, and it was clearly stated that leaving the group would carry no disadvantage. All 15 children provided parental consent and were included in the study.

The group was facilitated by two trained nutritionists from the research team (HJ and UK). Due to school system regulations, monetary incentives were not permitted. Instead, a field trip was organized at the end of the project to show appreciation for the children’s participation.

### Procedures and methods of active involvement to collaboratively develop an intervention

There are currently no guidelines for involving children in research available in the German-speaking area. We intended to apply one international guideline as an orientation to our project, e.g. ‘Involving children and young people in research: top tips and essential key issues for researchers by INVOLVE’ [22]. The ‘nutrition’ activity group met once a week (except during school-holidays) for 1.5 h over the course of 10 months. The meetings took place in a regular classroom at the school. We intentionally called the activity group ‘nutrition’ and left out terms like ‘healthy’ or ‘adequate’ to keep it more neutral and not to induce specific expectations. Table 1 shows the different phases of the collaboration process. These phases and the duration of the phases emerged from the process and were not planned beforehand.

**Table 1:** Phases of collaboration, the content and methods

Phase	Content	Method	Duration
Introduction	Introductions, aims, roles	Introduction rounds, discussions	2 meetings
Building relationships, Defining aims and roles	Getting to know each other, exchanging experiences and opinions	Discussions, mind maps	8 meetings
Collaboration process	Learning about the association of nutrition and health Components of the intervention (setting, target group, material, content, timing, duration)	Exposure to different foods and research methods, discussions Brainstorming, mind maps, time tables	6 meetings

During the first meeting, we explained to the children that our intention was to collaborate with them within a research team and we would like to involve them as experts of their own nutrition. For approximately the first three months, we avoided framing nutrition as “healthy” and instead opened discussions around everyday eating and drinking habits—what they eat, avoid, and why. We used interactive methods such as brainstorming and mind mapping.

The project had no fixed content or methods beyond the initial phase. Instead, the process evolved based on the children’s input and the demands of the research timeline, aiming to develop a scientifically sound and feasible intervention. No specific components were pre-defined, aside from the focus on dietary behaviour and that development would be guided by the children’s ideas.

As the sessions progressed, we introduced the concept of food groups, brainstorming and sorting foods together. In response to the children’s requests, we began each session by preparing healthy snacks. This phase, which lasted around 10–12 weeks, was crucial for team building and orientation.

After this phase of team building and orientation, we invited an external expert to introduce ‘healthy’ nutrition to us. She gave us an age-appropriate introduction into a healthy nutrition mainly by discussing a food pyramid [26]. The children learned about the variety of foods within each group and the recommended intake, fostering their interest in diet and nutrition. Following this, the research team—comprising the two researchers and the participating children—began developing the intervention components, carefully considering the information shared by the external expert. This process took place over the next two sessions through collaborative brainstorming, guided by two key questions:

1. Which tasty snacks align with our new knowledge of healthy nutrition?
2. How can we (the children) share what we have learned with our peers?

The material gathered from these two questions informed the design of the intervention over the following four to six sessions, leading to ideas such as offering snacks to peers and creating informative posters. The resulting intervention aimed to improve children’s dietary behaviour and was shaped by the children’s input, with researchers providing scientific guidance and ensuring feasibility. Early on, the children expressed a desire to implement the intervention in the school cafeteria by offering healthy food options to their peers. Although no setting was pre-defined, the children envisioned the school as the natural context. Ultimately, the intervention involved the weekly preparation and distribution of healthy snacks to younger students during school breaks. A detailed description of the intervention and its implementation is provided elsewhere [Klink U, Jilani H. Implementation and Evaluation of a Co-Developed Intervention to Promote Healthy Eating: Designed by Children for Children Journal of Prevention, Submitted.]. Lastly, we discussed and tested an instrument (e.g., Food Neophobia questionnaire) to evaluate the developed intervention.

Meetings generally started with a summary of the previous meeting, discussed collaboratively by children and researchers. Every member could contribute to the summary if they felt something was missing. For all phases and contents that we worked on we often used the method of brainstorming. For communication and saving our results we were able to use the online platform ‘itslearning’. This platform was used as a learning management system (LMS) by the whole school to organise lessons and assignments. Therefore, the children were very familiar with working on this platform. The same LMS is used by schools in Bremen as well as several other federal states in Germany.

The researchers introduced specific research methods and procedures during the activity group meetings to explain the rationale behind certain decisions and steps in the intervention development process. The children were introduced to data collection instruments, such as focus group discussions and questionnaires, used to gather both qualitative and quantitative scientific data. They were given the opportunity to test these instruments themselves, such as the Food Neophobia Questionnaire for children [27, 28]. Further, children received a “research journal” to record notes and reflections related to the group activities. Over the course of the semester, the children were assigned various practical tasks to support the project, including checking cafeteria opening hours and conducting interviews in their classes to gather preferences regarding food and beverage options.

In summary, after the first month, the entire process and content were developed collaboratively, allowing the project to remain flexible as it progressed. Finally, many ideas and contents could be collected and summarised into a feasible intervention for the school setting aiming at presenting healthy self-made snacks to children’s peers.

### Evaluation design and methods

A qualitative evaluation was carried out to examine the process of actively involving healthy children. Observational data were documented by the participating researchers through personal minutes and post-scripts of the activity group meetings. Additionally, a focus group discussion was conducted at the end of the intervention development phase. All data collection took place within the school setting. Detailed descriptions of the data collection procedures and analysis are provided in the subsequent sections. We followed the GRIPP2 checklist for reporting Public and Patient Involvement [29] which is available in the supplementary file 1.

### Collection of researchers’ and children’s perspectives on the active involvement

The researchers recorded written personal minutes during every meeting. Further, after the meetings HJ and UK noted additional observations in postscripts. Observations included factors like organisation, mood, needs, wishes, suggestions, contents of the meetings, and feedback.

After the development of the intervention, an audio-recorded semi-structured focus group discussion with a child-friendly method was conducted during a meeting of the activity group that lasted approximately 30 min. The instrument used in this study as an interview guide can be found in the supplementary file 2. The five finger method for feedback was used [30]. Here, a drawing of a hand with one question attached to each finger followed by an additional overarching question was shown to the children.

The questions used were:

1. What was great?
2. What did I learn?
3. What can be improved?
4. What do I take away (positive and negative)?
5. What was lacking?

And lastly: Anything else I want to say/What do I wish for?/Further suggestions?

Every question was first read out by one of the children. Then, time was given to answer and discuss every question. When the discussion was stopped, the researchers rephrased each question and asked again to remember the whole duration of the first part of the project to answer each question. The focus group discussion was led by the two researchers HJ and UK.

### Analysis

In order to answer our first research question, HJ and UK analysed the minutes and postscripts along the phases of the project. Both researchers first analysed their own minutes and postscripts and grouped the data into barriers and facilitators for active involvement for every phase of the project. Subsequently, a synopsis was written by HJ and discussed with UK. The phases were: ‘Initiating the process’, ‘Building relationships, defining aims and roles’ and ‘Collaboration process’.

The focus group discussion was recorded with a digital voice recorder and subsequently transcribed by an artificial intelligence program (Whisper AI). The University of Bremen has implemented Whisper AI as a tool for all employees and students on their server. The University recommends this tool for the transcription of interviews and states that it aligns with data protection policies. The transcripts were reviewed by UK and subsequently checked by HJ. Afterwards, all personally identifiable information such as names or other identifiable data was removed from the document. The transcript was read multiple times, categories for analysis were built inductively, data was coded, and analysed by HJ and UK independently according to the thematic qualitative text analysis suggested by Kuckartz [31].

Since the transcripts were in German, citations incorporated in this article were translated into English by HJ, checked by UK and then back-translated by a student assistant to verify the precision of the translations.

## Results

In total, 15 children (12 girls and 3 boys) enrolled in the ‘nutrition’ activity group. They were between 11 and 12 years of age. Approximately 8 children (6 girls and 2 boys) consistently attended and took part in the weekly meetings.

### Barriers and facilitators for active involvement from researchers’ perspective

#### Initiating the process

##### Barriers

The recruitment was perceived as challenging. Especially, due to the absence of established infrastructure and methodologies for the active involvement of the target group in research. Initial efforts in a church and a cultural organisation were unsuccessful. Ultimately, a school was identified as a suitable setting and agreed to participate. Nevertheless, in the school setting, we had difficulties to obtain written parental consent. We never got to meet the parents in person and had to hand out the study information several times via the children until all written consent forms were obtained.

##### Facilitators

Communicating the project’s aims to various stakeholders (e.g. headmaster, teachers, children) required particular attention, as the general public is typically unfamiliar with research processes, especially participatory approaches. We acknowledged that the headmaster of the school showed strong support, and a ‘nutrition’ activity group was successfully established. The headmaster ensured that all data protection requirements could be met, that a teacher was available as the official contact person for the activity group, that the researchers were granted access to its learning, and that all expenses for food could be reimbursed. Conducting the project within a familiar educational environment during regular school hours proved to be a facilitating factor for the research project.

#### Building relationships, defining aims and roles

##### Barriers

The initial phase focused on building trust, clarifying expectations, and establishing roles, which required approximately six weeks of weekly meetings, making it the longest phase of the project. Although the group’s objectives were introduced at the outset, students initially struggled to grasp the purpose of the activity. Misaligned expectations became apparent: children had selected the “nutrition” group anticipating cooking activities, while researchers aimed to collaboratively develop a healthy nutrition intervention. Children were unfamiliar with participatory research and the idea of collaboratively developing health programs with adults. In the school environment, children are typically treated as students by adults in authoritative roles, which made this shift challenging for both parties. Repeated discussions were necessary to establish a shared understanding, ensure the feasibility of ideas, and balance scientific requirements with children’s needs and preferences. We emphasised the importance of children’s input and committed to shared decision-making within reasonable constraints. Especially at the beginning of the activity group, the team was frequently engaged in discussions about common goals, expectations, and motivations of working together.

##### Facilitators

Clarifying mutual goals and redefining roles, particularly shifting from traditional teacher-student dynamics toward more collaborative partnerships, was very helpful and worth the effort. To foster involvement, each session began with a short check-in round to strengthen relationships and provide space for open expression. Discussions on dietary behaviour and food preferences revealed that children often felt hungry at school due to limited food offerings. In response, healthy snacks were introduced at meetings, serving both a practical and educational function. Preparing and tasting different foods helped make abstract concepts more tangible and supported a more interactive exploration of the topic.

#### Collaboration process

##### Barriers

While researchers had to communicate practical limitations (e.g., time, space, budget), children strongly advocated for the inclusion of hands-on food preparation and distribution in the school setting, emphasising what mattered most from their perspective. We as researchers had to navigate these limitations while preserving the children’s sense of ownership. The concept of “intervention components” was abstract and not inherently motivating for the children, often leading to topic shifts. As group dynamics evolved, dominant voices occasionally overshadowed quieter participants. To uphold inclusive involvement—a core principle of participatory research—the group was divided into smaller sub-groups, and alternative, child-friendly methods such as individual writing and drawing were used. These adjustments enabled broader involvement and collaboration.

##### Facilitators

Following the relationship-building phase, the development of the intervention marked the most intensive and collaborative stage. Collaboration required mutual respect, active listening, and continuous negotiation. Meaningful collaboration emerged primarily when discussions were rooted in the children’s everyday experiences. At the beginning of this phase, an external nutrition expert was invited (see procedure). This was perceived as very helpful, since although we were nutritional scientists as well, we felt it was not our role to teach the children in a teacher-student relationship. On this basis it was possible to develop the intervention.

Results of barriers and facilitators of each phase are summarised in Table 2.

**Table 2:** Barriers and facilitators of each phase when involving children actively in a research project

	Barriers for active involvement	Facilitators for active invovlement
Initiating the process	- No infrastructure and methods - Lack of awareness - Missing consent of parents	- Communication with all relevant stakeholders - Support of stakeholders
Building relationships	- Misaligned expectations and motives of children and researchers - Children not used to take decisions collaboratively - Determinations of roles	- Spending enough time and several meetings to find common objectives and compromises - Clarifying roles, goals and expectations as well as repeating to demonstrate the importance of everybody’s opinions - Check-in rounds - Planning snacks - Practical demonstrations
process of Collaboration	- Limited resources - Including wishes while simultaneously keeping scientific rigor - Keeping interest of the children - Diversity of participants	- Flexibility and willingness to compromise by all participants - Avoiding using scientific language - Including diverse instruments like smaller sub-groups, writing and drawing tasks (variety of methods) - Respect - Active listening - External expert - Considering children’s everyday experiences

### Results of the focus group discussion

The focus group discussion primarily explored feedback on the process of active involvement. As conversations among the children often drifted to unrelated topics, the researchers repeated each question and emphasized the collaborative aspect to encourage reflection and ensure clarity. The results can be grouped into three categories: ‘relationship and atmosphere’, ‘content relevance and core topics’ and ‘collaboration’.

#### Relationship and atmosphere

Most of the children appreciated the open and relaxed atmosphere, describing it as a pleasant way to unwind after a long school day. Some valued the freedom to be loud and not constantly expected to stay quiet. However, others expressed discomfort with the noise, particularly when more dominant peers became too loud during the sessions.

Student 1: “I really liked that I was allowed to be loud.”

Researcher 1: “Aren’t you usually allowed to? “.

Student 1: “No, not in my class — but at home, yes. “.

Student 4: “I loved that we got to do so much drawing. “.

The children appreciated that food was provided during the meetings, noting they were often hungry in the afternoon and enjoyed trying a variety of new foods.

Student 4: “It was great that we always tried out so many recipes and things like that. “.

The children enjoyed being able to paint and draw in the activity group and found it to be a time to relax, especially since they had a more demanding school subject before our meetings.

At the end, two of the children mentioned that they will always remember our relationship when they think of the working group and they wished it would never end:

Researcher 1: “What comes into your mind about what you have learned here? “.

Student 3: “I have learned that I should stay– for love. “.

Student 1: “…, the activity group is just, ehm, I would say…“.

Student 3: “… never stop! Never! In life! “.

#### Content relevance and core topics

Children mentioned learning about healthy nutrition and that it was better for their bodies. They specifically mentioned the food pyramid, the seasonality of fruits and vegetables and learning practical food skills such as cutting fruits and vegetables and preparing bread with spread and toppings.

Student 4: “It was great that we could always try so many recipes and so on. “.

Student 3: “Yes. That, ehm, that we before the, after classes, we were hungry and we could eat here. “.

Student 4: “That it was directly after GUP *[demanding school subject]*, because here it was relaxed. “.

Student 3: “That healthy food, eh, ehm, is better for our bodies. “.

Student 4: “Ehm, the food pyramid, that is what we learnt. “.

Student 4: “We have learnt which fruits and vegetables grow in which year, eh, month. “.

When asked what they would remember about the activity group, the children mentioned taking away many positive insights about healthy nutrition. They especially enjoyed the visit from the external expert who explained the food pyramid and different food groups, as well as the day cake was brought in. One child shared that they had started paying more attention to their diet and were making healthier food choices as a result.

Researcher 1: “Yes. What do you think of when you think of our working group? “.

Student 3: “That I ate very little healthy food. From now on? I always eat healthy. “.

The students expressed a desire for more time to play games, paint, and draw, as well as for another visit from the expert. They also shared wishes for future activities, particularly the opportunity to cook or bake and to carry out something in the school cafeteria.

Researcher 2: “What could be better? What would you wish for? “.

Student 3: “Nothing– that we start cooking. “.

Student 2: “That we do a competition [in preparing foods and snacks]. “.

When asked what could be improved in the activity group, explicit desires for future activities were mentioned, particularly cooking and a “challenge.” Other mentions included more worksheets (which was strongly rejected by other students), bringing in muffins to decorate, and shortening the duration of the working group.

#### Collaboration

Generally, the children appreciated the interactive collaboration and the relatively free and open setting.

It was clear that some children greatly enjoyed the activity group, with one referring to it affectionately as “club love.” While several children were very outgoing during the feedback session, two were more reserved and only responded when directly addressed. One child with special needs had difficulty understanding the questions and recalling their thoughts but was able to contribute when the questions were clarified. Table 3 summarises the results of the focus group discussion and adds the observations of the researchers regarding children’s feedback after several follow-up questions.

**Table 3:** Summary of the focus groups discussion

Identified Categories	Summary children’s feedback	Additional observations by researchers
Atmosphere	- Appreciation of open and pleasant atmosphere while preparing and eating (new) foods	- Teambuilding was not mentioned
Content relevance and core topics	- Learnings about healthy eating - Food pyramid - Seasonality of fruits and vegetables - Practical food handling - Wished for baking and cooking	- Despite several follow-up questions, none of which explicitly addressed the intervention, children did not mention the development of the intervention, which was the overarching aim of the project
Collaboration	- Liking the interactive arrangement of the meetings - Recognition that they were able to do what they wanted	- Although researchers asked several times and observed during the meetings that children appreciated taking decisions, children did never mention the true collaboration explicitly.

## Discussion

### Main results

This investigation aimed to answer two research questions; [1] What are the barriers and facilitators from the researchers’ perspective when involving healthy children in research? and [2] How do children perceive their active involvement in developing a healthy nutrition intervention in Germany? Researchers noticed that a high degree of flexibility and time resources are needed to actively involve children in a research project. Initiating the project as well as building relationship required a high amount of time and effort before the real collaboration process could even start. The primary findings of the evaluation indicated that children enjoyed being interactively and practically involved in the project.

### From barriers and facilitators to implications for future projects

Our results led to some learnings and implications for future projects. First of all, it is important to be flexible in terms of content and aims to make the active involvement of children meaningful, as children may have vastly different perspectives and interests that vary from those of the researchers and have different approaches to grasp a subject. Further, sufficient time should be planned for building relationships and discussing shared goals and roles, as children may need additional support and time to express their thoughts. Across various settings and conditions, studies have consistently highlighted the importance of dedicating adequate time and establishing clear agreements on aims and roles [32, 33]. Our results and implications are in line with prior published results such as addressing power imbalances and the need for flexibility [25]. Further, building strong relationships between children and researchers contributes to the success of such an undertaking. This leads to another learning that the children should be regarded as experts for their own needs and perspectives as also highlighted by ‘The International Charter for Ethical Research Involving Children’ [23]. In general, it is important to be aware of the expertise of every member within the collaboration process since this is connected to the role that someone can fulfil within the team.

Especially during the establishment and initiation of the active involvement we faced several practical difficulties. This should be considered when planning collaborations with the target group, especially when they are minors. Ethical approval, organisation, communication and logistical details can be time consuming. When working with minors the parents have to give consent and therefore need to be involved before it is even possible to start.

It is advisable to inform children very thoroughly about what they should expect before choosing to participate. To increase the number of children signing up for the activity, researchers could visit the setting beforehand and provide information about the activity. Again, this demands time. Nevertheless, it prevents children from being disappointed for having had expectations that cannot be fulfilled.

Another challenge can be the diversity of the children. Some might be louder, some more quiet. The team should take care of each child involved and motivate everyone to be and remain engaged and committed. As reported in the results, in this study, different methods were applied to take care of every child’s personality and capacities. On the one hand, it is important to involve diverse personalities and backgrounds in order to consider different perspectives. On the other hand, this requires more effort along the whole process to keep every team member committed. In future projects, new combinations of methods can be tested to enhance attractiveness to the children and to involve more children. Brady and Preston (2020) highlighted the importance of involving children from diverse backgrounds, acknowledging this diversity, and fostering open dialogue about the impact of children’s involvement [34]. Moreover, one way to foster active involvement is by enhancing researchers’ capacities and expertise in this area [35]. Rouncefield-Swales et al. (2021) conducted a scoping review on the involvement and engagement of children and young people. They concluded that researchers should adopt a pragmatic and flexible approach to participation, and that children’s preferences, choices, abilities, and interests are taken seriously and meaningfully integrated into the research process [36]. Thus, involving the target group in research creates a mutual learning process, where both researchers and participants learn from one another, as also emphasized in earlier research [37].

**Table:** Box 1: Key recommendations for future projects involving healthy children in research

✥ Be flexible in terms of aims and content to enable meaningful involvement
✥ Take time to build relationships and trust as well as to discuss roles and goals
✥ Consider children as experts for their own needs and perspectives
✥ Be transparent from the beginning to avoid disappointment
✥ Involve children with diverse backgrounds and take care of the diversity

### Level and intensity of involvement

The goal of active involvement in this project was to jointly develop a healthy nutrition intervention through a collaborative, child-led process. Both the approach and content were intentionally left open, allowing children to shape the process meaningfully. As a result, the procedure and the final intervention were developed in close partnership with the children. Based on the levels of involvement defined in the introduction (ranging from consultation to collaboration to user-led research) this project aligns with consultation, approaching collaboration, primarily because final decision making for some components remained with the researchers.

We chose collaborative and intensive methods of involvement, meeting with the entire group for 1.5 h each week over 10 months. To ensure meaningful participation from each child, we limited the group to those who initially signed up (*n* = 15). While we aimed to involve children from diverse backgrounds, our original plan to implement the project within a community-based setting, such as a church or cultural center, proved unfeasible due to practical barriers. Such settings would have allowed access to children from various neighborhoods and sociodemographic backgrounds. Ultimately, we adapted the project to the conditions of our school partner, which shaped many aspects of the process. For instance, while weekly meetings were not originally intended, they proved to be the most feasible option within the school context and children’s daily routines.

The introductory phase, focused on building relationships, required considerable time but proved to be the most crucial stage of the project. Although this period did not directly involve developing the intervention, it laid the foundation for effective collaboration and meaningful participation. Similarly, a study from the Netherlands highlighted that establishing relationships with key stakeholders, such as schools and children, is time-intensive yet essential for the success of participatory projects [38].

### Roles and relationships

In a context where children are accustomed to hierarchical relationships with adults—especially in school—significant effort was required to explain the project’s aims and collaborative approach. The children were unfamiliar with having their perspectives valued to such an extent. While focus group results showed that they clearly remembered the session content and felt their input was taken seriously, they did not reflect on their role as research team members or the broader goals of the project. Despite researchers’ efforts to explain each stage, the overarching purpose of the collaboration remained abstract to the children. As active involvement in research becomes more publicly recognized and supported by clearer communication and recruitment methods, it may become easier for children to engage with and understand these aims. Currently, a lack of public awareness, unclear terminology, and inconsistent usage of existing terms continue to impede the broader adoption of participatory approaches. Developing and consistently applying standardized definitions is therefore essential [39]. In future projects, new combinations of methods can be tested to enhance attractiveness to the children and to involve more children. The number of children involved per group [38, 40] and the methods used [40, 41] in former projects were similar to our study while other studies also used more diverse methods such as photovoice and online methods [42]. Nevertheless, when using photovoice and online methods, data protection issues have to be considered.

### Strengths and limitations

This study holds several strengths. Our setting enabled intensive collaborative work with the children. Due to our weekly meetings we were able to build a strong relationship with the children. The contents we discussed were not forgotten between the meetings and we could continue without a long phase of repetitions. Further, this particular project was equipped with time, flexibility as well as monetary and personal resources to enable meaningful participation. Our approach enabled a high degree of flexibility regarding setting and content of the intervention since we had no predefinition regarding these parameters.

Beside these strengths, the study had several limitations. Due to practical reasons our project was conducted in a school. This enabled only children that were enrolled in this school to participate in our project. The requirement of the school that it offered activity groups only for sixth-graders limited our participants to children between 11 and 12 years of age. Thus, future projects that involve a wider range of age-groups are needed to describe collaboration across different age-groups. Nevertheless, we were able to include children of both sexes and with and without migration background. Further, the school setting fostered the anticipation among the children that we would have a teacher-student relationship within our working group. Possibly, the traditional power structures within school settings are maintaining a top-down approach in the development of the intervention [16]. Consequently, it took a lot of effort to build a team that works together on an equal footing. Another review focusing on communication processes during collaboration with children suggested that, in participatory projects, children need a new understanding of adults. This helps reduce the power gap and enables them to feel like full members of the research team [43]. The high frequency of meetings required greater organisational effort. However, it also contributed to the previously mentioned strengths of the process, e.g., a strong relationship.

A potential limitation of this study is that the same individuals were involved in both the implementation and the evaluation of the process. This dual role may have introduced bias, as objectivity could be limited or children might have responded in a socially desirable manner due to the evaluators’ involvement in the project itself. However, this close involvement also allowed for a deeper contextual understanding and flexible, process-oriented data collection. A reflexive approach, including regular reflection on roles and transparent reporting, was applied to mitigate potential bias. Further, participating children had already established an open and trusting relationship with the researchers, which made it ethically, legally (in terms of data protection), and contextually inappropriate to involve external evaluators.

## Conclusion

Through the qualitative evaluation of the active involvement this study highlights the importance of actively involving children in health research through flexible and participatory methods, emphasising the need for time, resources, and adaptability to ensure meaningful involvement. Establishing strong relationships between researchers and children is a crucial step, as it fosters mutual understanding and sets the foundation for effective teamwork, and therefore, the successful development of interventions. Despite practical challenges such as limitations of school-based settings, participant diversity, and the traditional power dynamics between adults and children, the project demonstrated the value of treating children as experts in their own needs and perspectives. The findings underline the significance of thorough communication with both children and their parents, clear expectations, and tailored methods to address the varying preferences and involvement levels of participants. Future projects should aim to include diverse age groups, explore innovative participatory tools, and continue refining methods to bridge power gaps, ensuring children feel valued as active collaborators in research. By describing and evaluating the involvement of healthy children in research, this investigation offers valuable insights for researchers planning to actively involve healthy children in research projects, helping to facilitate implementation and reduce potential barriers.

## Supplementary Information

Supplementary Material 1.

Supplementary Material 2.

## Data Availability

The datasets used and analysed during the current study are available from the corresponding author on reasonable request.
