# Supplementary material for: Active involvement of children aged 11–12 years in the development of a healthy nutrition intervention – a qualitative evaluation from researchers’ and children’s perspectives
Source: BMC Public Health. 2025 Aug 14;25:2767. doi: 10.1186/s12889-025-24019-x (PMC12351763; doi:10.1186/s12889-025-24019-x)
Supplement: Supplementary file 1 — Supplementary Material 1. [file 12889_2025_24019_MOESM1_ESM.pdf]

Supplementary file 1: GRIPP 2 checklist

Section and topic	Item	Reported on page No
**Section 1: Abstract of paper**		
1a: Aim	Report the aim of the study	3
1b: Methods	Describe the methods used by which patients and the public were involved	3
1c: Results	Report the impacts and outcomes of PPI in the study	3
1d:Conclusions	Summarise the main conclusions of the study	3
1e: Keywords	Include PPI, "patient and public involvement," or alternative terms as keywords	1
**Section 2: Background to paper**		
2a: Definition	Report the definition of PPI used in the study and how it links to comparable studies	5
2b: Theoretical underpinnings	Report the theoretical rationale and any theoretical influences relating to PPI in the study	5
2c: Concepts and theory development	Report any conceptual models or influences used in the study	5
Section 3: Aims of paper		
**3: Aim**	**Report the aim of the study**	
**Section 4: Methods of paper**		
4a: Design	Provide a clear description of methods by which patients and the public were involved	7-11
4b: People involved	Provide a description of patients, carers, and the public involved with the PPI activity in the study	7-8
4c: Stages of involvement	Report on how PPI is used at different stages of the study	8-11
4d: Level or nature of involvement	Report the level or nature of PPI used at various stages of the study	8-11
**Section 5: Capture or measurement of PPI impact**		
5a: Qualitative evidence of impact	If applicable, report the methods used to qualitatively explore the impact of PPI in the study	11-12
5b: Quantitative evidence of impact	If applicable, report the methods used to quantitatively measure or assess the impact of PPI	n.a.

5c: Robustness of measure	If applicable, report the rigour of the method used to capture or measure the impact of PPI	n.a.
**Section 6: Economic assessment**		
6: Economic assessment	If applicable, report the method used for an economic assessment of PPI	n.a.
**Section 7: Study results**		
7a: Outcomes of PPI	Report the results of PPI in the study, including both positive and negative outcomes	13-20
7b: Impacts of PPI	Report the positive and negative impacts that PPI has had on the research, the individuals involved (including patients and researchers), and wider impacts	13-20
7c: Context of PPI	Report the influence of any contextual factors that enabled or hindered the process or impact of PPI	13-20
7d: Process of PPI	Report the influence of any process factors, that enabled or hindered the impact of PPI	13-20
7ei: Theory development	Report any conceptual or theoretical development in PPI that have emerged	20-22
7eii: Theory development	Report evaluation of theoretical models, if any	20-22
7f: Measurement	If applicable, report all aspects of instrument development and testing (eg, validity, reliability, feasibility, acceptability, responsiveness, interpretability, appropriateness, precision)	n.a.
7g: Economic assessment	Report any information on the costs or benefit of PPI	n.a.
**Section 8: Discussion and conclusions**		
8a: Outcomes	Comment on how PPI influenced the study overall. Describe positive and negative effects	20-25
8b: Impacts	Comment on the different impacts of PPI identified in this study and how they contribute to new knowledge	23-25
8c: Definition	Comment on the definition of PPI used (reported in the Background section) and whether or not you would suggest any changes	20-21
8d: Theoretical underpinnings	Comment on any way your study adds to the theoretical development of PPI	25

8e: Context	Comment on how context factors influenced PPI in the study	20-23
8f: Process	Comment on how process factors influenced PPI in the study	20-23
8g: Measurement and capture of PPI impact	If applicable, comment on how well PPI impact was evaluated or measured in the study	20-25
8h: Economic assessment	If applicable, discuss any aspects of the economic cost or benefit of PPI, particularly any suggestions for future economic modelling.	n.a.
8i: Reflections/critical perspective	Comment critically on the study, reflecting on the things that went well and those that did not, so that others can learn from this study	22-25
