# Supplementary material for: Active involvement of children aged 11–12 years in the development of a healthy nutrition intervention – a qualitative evaluation from researchers’ and children’s perspectives
Source: BMC Public Health. 2025 Aug 14;25:2767. doi: 10.1186/s12889-025-24019-x (PMC12351763; doi:10.1186/s12889-025-24019-x)

Supplementary file 2: The child-friendly five finger method for feedback

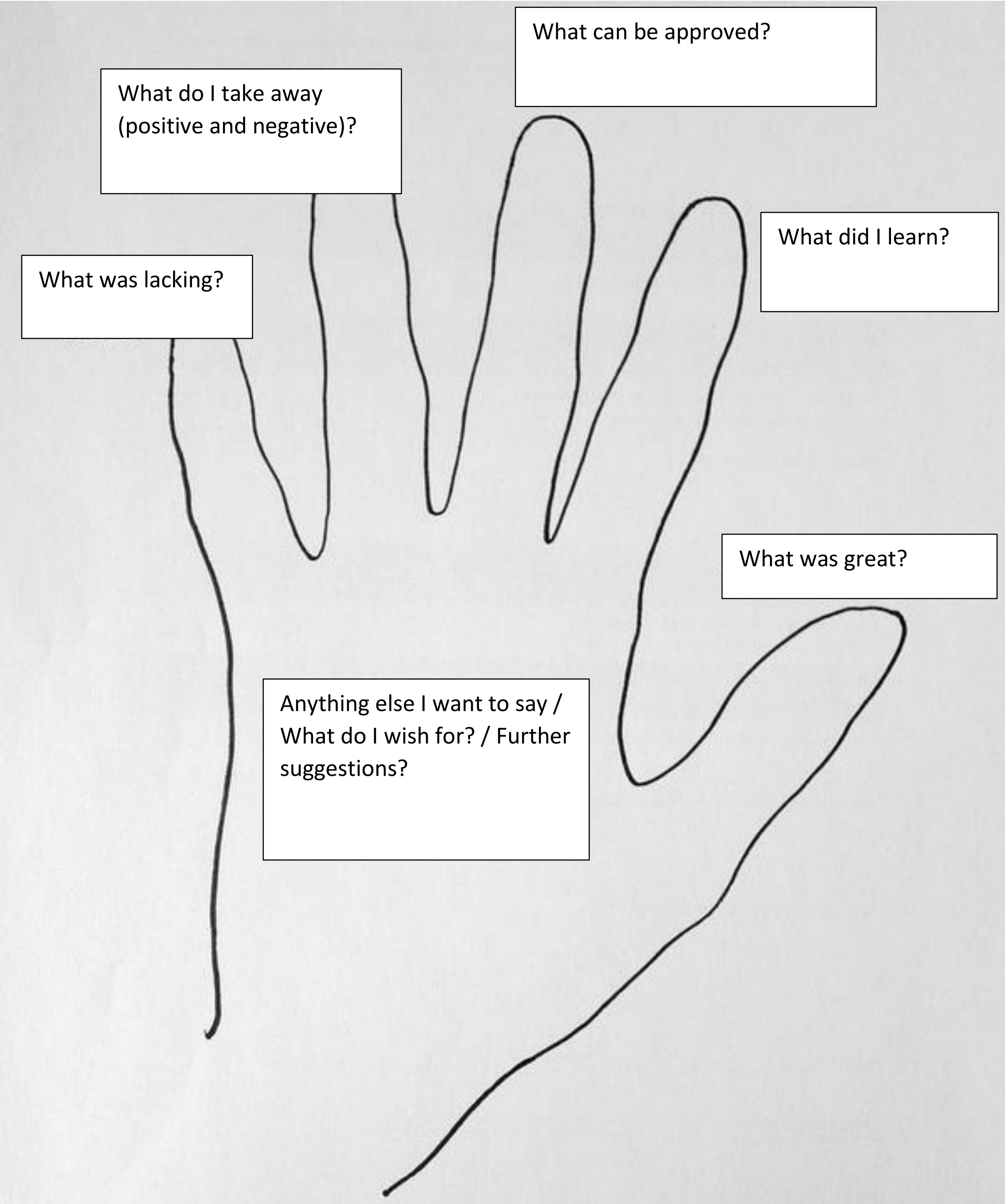

Supplement: Supplementary file 2 — Supplementary Material 2. [file 12889_2025_24019_MOESM2_ESM.pdf]
